# Novel insights into adipose tissue heterogeneity

**DOI:** 10.1007/s11154-021-09703-8

**Published:** 2021-12-21

**Authors:** Tongtong Wang, Anand Kumar Sharma, Christian Wolfrum

**Affiliations:** Institute for Food, Nutrition, and Health, ETH Zurich, Schorenstrasse 16, Schwerzenbach, 8603 Switzerland

**Keywords:** Single cell RNA sequencing, Adipose tissue, Heterogeneity, Mature adipocytes, Stromal vascular fraction

## Abstract

When normalized to volume, adipose tissue is comprised mainly of large lipid metabolizing and storing cells called adipocytes. Strikingly, the numerical representation of non-adipocytes, composed of a wide variety of cell types found in the so-called stromal vascular fraction (SVF), outnumber adipocytes by far. Besides its function in energy storage, adipose tissue has emerged as a versatile organ that regulates systemic metabolism and has therefore constituted an attractive target for the treatment of metabolic diseases. Recent high-resolution single cells/nucleus RNA seq data exemplify an intriguingly profound diversity of both adipocytes and SVF cells in all adipose depots, and the current data, while limited, demonstrate the significance of the intra-tissue cell composition in shaping the overall functionality of this tissue. Due to the complexity of adipose tissue, our understanding of the biological relevance of this heterogeneity and plasticity is fractional. Therefore, establishing atlases of adipose tissue cell heterogeneity is the first step towards generating an understanding of these functionalities. In this review, we will describe the current knowledge on adipose tissue cell composition and the heterogeneity of single-cell RNA sequencing, including the technical limitations.

## Introduction

The identification of adipose as an important place for energy storage in humans can be traced back to Medieval times, and this view has persisted up until recently. Since the identification of leptin [1, 2], adipose tissue has been progressively recognized as a dynamic organ with many and varying function in the regulation of whole body metabolism and systemic energy homeostasis [3]. The prevalence of obesity, characterized by excessive accumulation of lipids in the adipose tissue [4], is rising worldwide. Given the unquestionable and major risk represented by obesity for a number of chronic disorders (including but not limited to type 2 diabetes, cardiovascular diseases, inflammation, and cancer [5]), adipose tissue has received renewed interest in recent years as a physiologically important organ and a therapeutic target. Adipose tissue responds rapidly to a change in the nutritional status and, in times of nutrient excess, acts as an energy sink. A chronic positive energy balance is controlled through adipocyte hypertrophy (increase in cell size) and hyperplasia (increase in cell number) in order to maintain lipid storage and thus regulate systemic metabolism [3]. During nutritional scarcity, adipose tissue mobilizes stored lipids to fulfill systemic energy demands. Therefore, the recruitment and differentiation of new adipogenic progenitor cells (APCs) in white adipose tissue offers protection from obesity-associated metabolic complications, by improving lipid storage and protecting the body from an excess carbon load [6].

Given the central role of adipose tissue in energy homeostasis, nutrient response, and association with metabolic disease, a better understanding of adipose cell biology may lead to novel strategies of targeting obesity or its metabolic co-morbidities [7]. Recent studies have highlighted the cellular heterogeneity of adipose tissue and its implication in functional outcomes. Therefore, it is essential to establish a comprehensive map of adipose tissue cellular components and to subsequently define mechanisms of intercellular communication that mediate pathological and/or therapeutic responses [8].

## Cellular heterogeneity confers functional versatility

Functionally, adipose depots are usually classified as brown or white adipose tissue. Brown adipocytes contain multiple small lipid droplets and a high density of mitochondria with the hallmark uncoupling protein 1 (UCP1) expression [9]. In contrast, classical white adipocytes, which store lipids [3], usually exhibit a single lipid droplet and a low mitochondrial density. As convincing as this simplistic view might be, it is well known that adipose tissue depots do not contain a homogenous cell population. It is also understood that adipocytes within a single adipose depot exhibit great morphological and functional diversity. One example of this is the identification of UCP1 positive brite/beige adipocytes within normally white adipose tissue [10, 11].

**Fig. 1:**
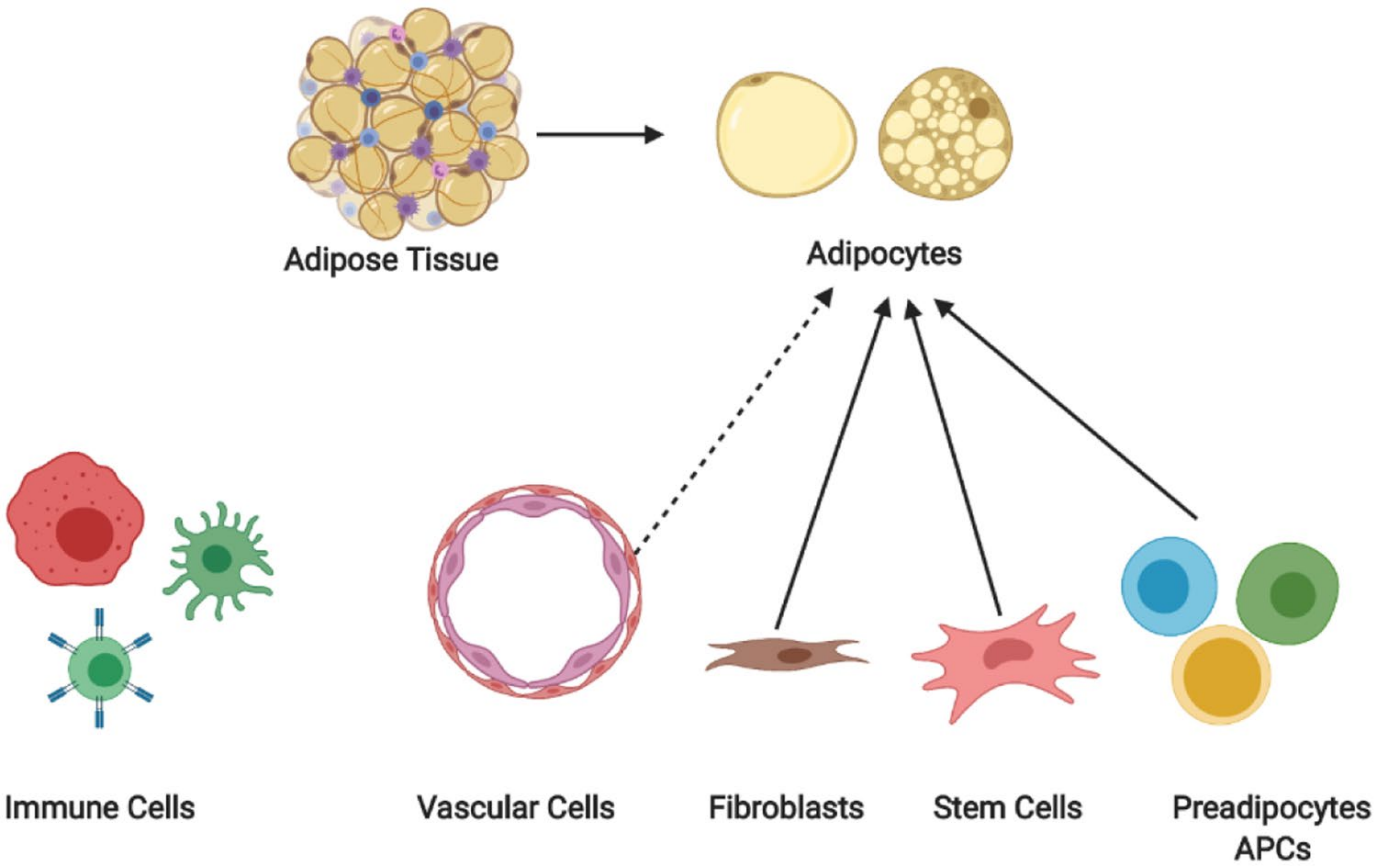
Schematic overview of the heterogeneity of the adipose tissue

Besides the mature adipocyte fraction, the heterogeneity of the stromal vascular fraction (SVF) has also received substantial interest in recent years. Mature adipocytes constitute only ~15 ~30% of the total adipose cell fraction, while the rest is classified as the SVF ( \* MERGEFORMAT Fig. 1), which consists of many different cell types, including immune cells, fibroblasts, vascular cells, APCs, and stem cells [12–14]. Besides APCs, other SVF cell populations directly interact with APCs/adipocytes and regulate both adipocyte fate and function. In conclusion, the cellular heterogeneity of mature adipocytes, along with that of cells within the SVF of adipose tissue, can be considered one of the major determinants of adipose tissue function and plasticity. In the following section, we will discuss studies that have aimed at decoding the cellular heterogeneity of adipose tissue and outline our current understanding of the dynamics of different cell populations as a result of genetic/environmental manipulation.

## Identification of cell heterogeneity in pre-single cell sequencing era

Early studies, which have aimed to identify APCs from total white adipose tissue, used SVF combined FACS and lineage tracing to discern and characterize previously unknown cell subpopulations. In a pioneering study, Berry et al. demonstrated that APCs are derived from the Lin^−^ lineage and express a set of surface markers (Cd34^+^, Cd29^+^, Sca1^+^, Cd24^+^) that can be used to reliably enrich this adipogenic population [12]. In further studies, the group also demonstrated that members of the PDGFRα^+^ mesenchymal cell population efficiently differentiate to adipocytes [15]. Later studies demonstrated that, within the adipose tissue, vascular cells could be the source of APCs [16], and that the adipose vasculature is the main site of the recruitment/commitment of new APCs [13]. In particular, smooth muscle cells and pericytes (labeled by SMA, NG2, SM22 [14], and PDGFRb [17]) have demonstrated their ability to give rise to adipocytes under various conditions. These studies, utilizing a limited number of surface markers, project underappreciated cellular diversity within APCs and were essential in defining the adipogenic populations. Nonetheless, these studies also retain a degree of ambiguity. For instance, high-specificity markers for APCs have not been identified thus far [18], and most of the populations referred to above seem to constitute an enrichment of the “true” APCs population. Additionally, the origin of APCs is still a matter of debate, due to the inherent problems associated with lineage tracing *in vivo*. Lastly, the functionality of the various APC and adipocyte populations has not been fully elucidated. Therefore, it is critical to uncover the heterogeneity of APCs and adipocytes in adipose tissue and explore their function.

## Emergence of single cell/nucleus RNA sequencing and its application to decode adipose tissue heterogeneity

Single cell RNA sequencing (scRNAseq), which can be defined as a next generation, high-frequency throughput RNA, sequencing of thousands of cells at the resolution of each individual cell, has enabled the transcriptomic analysis of cell heterogeneity, state, and dynamics. In recent years, scRNAseq has become much more effective and economically affordable. Therefore, applying single cell approaches doesn’t only help to overcome the averaging artifacts associated with traditional bulk sequencing data, but also enhances our understanding of the cellular heterogeneity underlying superficially homogeneous populations [19, 20].

**Fig. 2:**
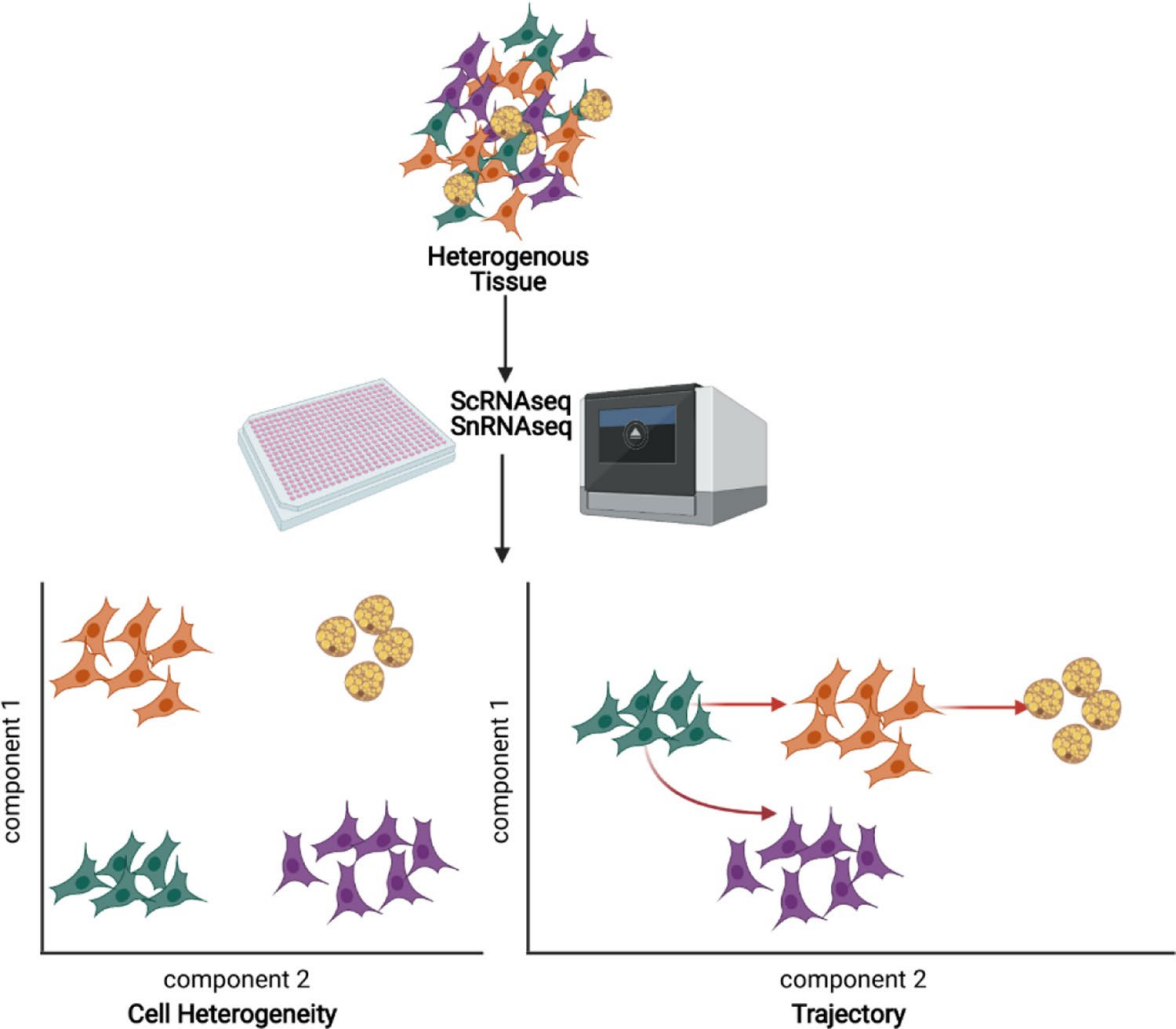
Schematic overview on single cell/nucleus RNA sequencing technologies

Single nucleus sequencing (snRNAseq) is a modified version of scRNAseq, wherein a single nucleus is sequenced for actively transcribed RNA instead of a single cell. This technique is especially effective on tissues that cannot be dissociated into intact single cells, such as adipocytes. Moreover, snRNAseq is also well suited to frozen samples that cannot be processed for scRNAseq [21]. Thus far, scRNAseq and snRNAseq have already demonstrated great success in unraveling complex cell populations, reconstructing developmental trajectories, and modeling transcriptional dynamics [20]. Several studies utilizing these technologies have explored adipose tissue cell heterogeneity ( \* MERGEFORMAT Fig. 2). The following section briefly compiles the current knowledge of the main identified APCs and adipocyte populations.

## Heterogeneity of Lin^−^ progenitor cells from inguinal adipose tissue

**Fig. 3:**
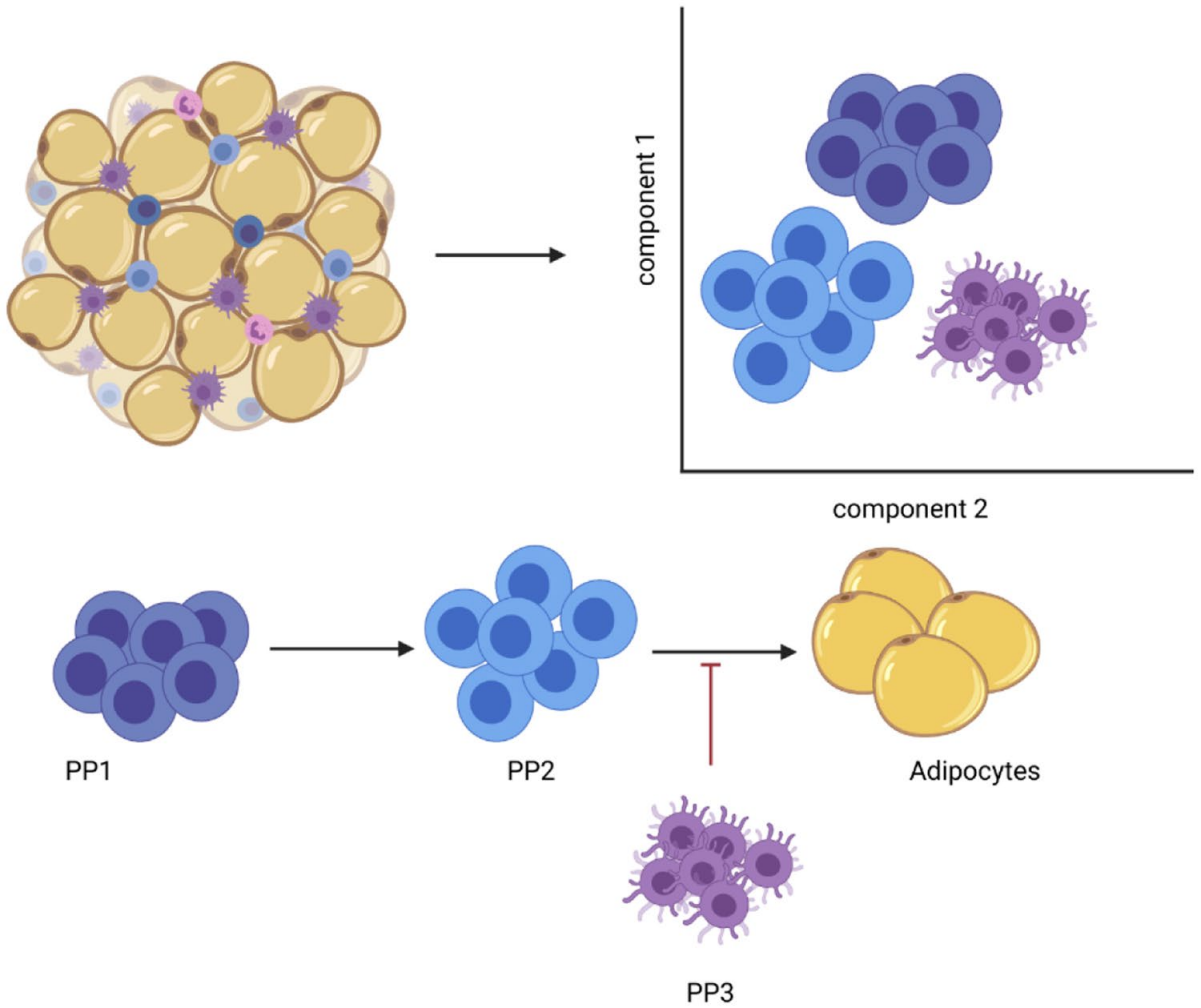
Schematic overview of the heterogeneity of the Lin- progenitor cells from inguinal adipose tissue

In many tissues, the Lin^−^ population of cells [11] constitutes an enriched fraction of multipotent stem cell/progenitor cells, including the APCs. Lin^−^ cells from the inguinal adipose tissue of mice were categorized by FACS and scRNAseq, which led to the identification of three APCs populations: (i) Dipeptidyl peptidase–4 expressing (i.e. Dpp4^+^) progenitor population (PP1), (ii) Intercellular adhesion molecule–1 expressing (Icam1^+^; but Cd142^−^) population (PP2), and a (iii) Cd142^+^ population (PP3) [22]. Each of these cell populations were characterized for either their adipogenic potential or the regulation thereof, which demonstrated the presence of highly proliferative, multipotent progenitors (PP1) residing in the reticular interstitium. Tracing studies demonstrated that, during subcutaneous adipose tissue development, PP1 cells give rise to PP2 committed preadipocytes and PP3 preadipocytes. This process is tightly controlled by transforming growth factor–β (TGFβ), which maintains cell identity by inhibiting the adipogenic commitment of PP1 cells [23] ( \* MERGEFORMAT Fig. 3). In a study performed by us, PP3 cells, which reside in the perivascular sheath, were identified as adipogenesis-regulatory cells that can suppress adipocyte formation through a paracrine regulation involving the secretion of key factors such as Spink2, Rtp3, Vit, and/or Fgf12. Interestingly, the PP3 cell numbers were found to be higher in obesity, suggesting the importance of these cells in metabolic control, insulin sensitivity, and type 2 diabetes [20]. Newer studies demonstrate that PP3 cells are not limited to adipose tissue but can also be found in muscle where they exert paracrine anti-adipogenic effects through the action of Gdf10 [24]. Taken together, these studies demonstrate the immense heterogeneity of the APCs populations. Furthermore, they show that the process of adipocyte formation is influenced by APCs commitment and regulatory cells, thereby regulating adipose tissue function as well as systemic metabolism.

## Heterogeneity of vascular mural APCs

Adipose tissue resident APCs and preadipocytes are undoubtedly important source of newly formed adipocytes. Moreover, as mentioned above, adipocytes possibly originate from vascular smooth muscle cells and pericytes, collectively known as mural cells. Because of the discrepancies in the methodologies utilized, controversy surrounding the research of mural cells has persisted for several years. Recently, scRNAseq has been introduced to address vascular mural APCs and their function ( \* MERGEFORMAT \* MERGEFORMAT Table 1). In their early efforts to determine SVF heterogeneity via methods of targeted identification and isolation of functionally distinct subpopulations, Hepler et al. performed an scRNAseq of PDGFRb + stromal vascular mural cells within the visceral WAT of adult mice [25]. They identified two distinct populations of cells. One population was marked by LY6C^−^/CD9^−^/PDGFRb^+^ representing a highly adipogenic visceral APCs. Another cluster of PDGFRb^+^ cells expressed LY6C (LY6C/PDGFRb double positive cells) and was subsequently named Fibro-inflammatory progenitors (FIPs), which lack adipogenic capacity, display a pro-fibrogenic/pro-inflammatory phenotype, and negatively regulate the adipogenesis of existing APCs. Similarly to the above-mentioned work regarding the subcutaneous tissue, these findings contribute to our understanding of the functional heterogeneity of visceral WAT perivascular cells [22]. When analyzed in a combined manner, it seems to be the case that the general principle of regulatory cells and a strict hierarchy of APCs are common denominators of both depots, even though the identified populations in subcutaneous and visceral adipose tissue are quite different.

**Table 1:** Vascular Mural APCs

Vascular Mural APCs	Markers	Function
LY6C-/CD9-/PDGFRb +	LY6C-/CD9-/PDGFRb	high adipogenic capacity
FIPs	LY6C/CD9-/PDGFRb	negatively regulate adipogenesis
CD81 +	CD81	precursors for brite/beige adipocytes
MYH11 + ; PDGFRA − ; PPARG +	Myh11; Pdgfra − ; Pparg	Precursors for brown adipocytes

As a result of research done in the last year, these studies have been extended to include the heterogeneity of white and brite/beige adipocytes. Using scRNAseq, Oguri et al. identified a novel, CD81^+^ APCs population that displayed a gene expression typical of vascular smooth muscle cells present in inguinal, visceral, and brown adipose tissue. More importantly, CD81^+^ cells respond to the cold challenge by giving rise to new brite/beige positive adipocytes in the inguinal adipose tissue, while the loss of CD81 causes diet-induced obesity and other complications [26]. In the newest study, Angueira et al. reported three brown adipogenic populations by applying scRNAseq and linage tracing to perivascular adipose tissue, comprising progenitors (Pdgfra + ; Ly6a + ; Pparg-), preadipocytes (Pdgfra + ; Ly6a-; Pparg +) [27], and a novel subpopulation of smooth muscle cells (Myh11 + ; Pdgfra − ; Pparg +) within the aortic adventitia of adult mice. These three populations of cells possess the capacity to generate adipocytes *in vitro* and *in vivo* and give rise to brown adipocytes in perivascular adipose tissue.

Taken together, these studies demonstrate a vascular lineage of pre-adipocyte or APCs and thus expand our understanding of the process of adipogenesis, as well as a hierarchy of APCs and their cellular fate. Additionally, these insights will be critical towards the development of future methods of metabolic targeted therapy. For example, CD81^+^ mural cells found in inguinal adipose tissue, as well as the adipogenic smooth muscle cells (Myh11 + ; Pdgfra-; Pparg +) of perivascular adipose tissue, might be utilized to promote the formation of brite/beige/brown adipocytes. Similarly, the identified FIPs constitute another promising cell population for therapeutic targeting, as they exhibit a fibrosis molecular signature and display a pro-inflammation phenotype, both of which are hallmarks of unhealthy adipose tissue expansion.

## Heterogeneity of APCs under different physiological and pathophysiological conditions

In an obesogenic/diabetogenic environment, macrophages have been reported to infiltrate adipose tissue and orchestrate a coordinated pro-inflammatory response, causing chronic inflammation [28]. It is believed that this process skews the balance from adipocyte hyperplasia to adipocyte hypertrophy [29], thus contributing to impaired metabolic health. Given that adipocyte hypertrophy is considered a pathological expansion (while adipocyte hyperplasia exerts protective effects), the inability to recruit new adipocytes might predispose subjects to obesity associated metabolic disorders [30]. To explore this notion, Vijay et al. conducted a study where CD45^−^ CD31^−^ CD34^+^ APCs from healthy, obese/diabetic humans were analyzed via scRNAseq. The authors found that two putative APCs populations, which are termed hP1 in subcutaneous and hP4 in visceral adipose tissue and express GPX3, ATF3 and ADH1B, are associated with the Type 2 diabetes (T2D) status. Similarly, two other populations (hP4 in subcutaneous and hP5 in visceral adipose tissue) expressed genes involved in fibrosis and extracellular matrix accumulation [31]. Aside from the depot-redundant cell populations, two populations, namely hP2 and hP3, were found to be subcutaneous, depot-specific APCs. hP3 cells expressed adipose stem cell markers while hP2 cells showed signatures of more mature adipocytes: APOE, FABP4, and CEBPB. Lastly, three populations (hP1-3) were identified as visceral, depot-specific progenitor populations, which have the ability to form brite/beige adipocytes [31]. The reported association of T2D incidence with the abundance of hP1 and hP4 in subcutaneous and visceral depots, respectively, is of high interest, as these cell types are characterized by lower gene expression of PPARγ, which has been reported to play a protective role in the development of insulin resistance [32]. Thus, targeting these APC subtypes could be a novel strategy to treat T2D. Also, hP4 and hP5 in subcutaneous and visceral adipose tissue, respectively, share inflammatory and fibrotic phenotypes similar to those reported for FIPs, indicating that inflammation and fibrosis-related constituents of adipose tissue exist in various species.

Besides the studies related to obesity and T2D, several studies have focused on the plasticity of cell populations in response to β3-adrenergic receptor activation, which results in brite/ beige adipocyte recruitment [10, 11]. When adipogenesis in inguinal and epididymal adipose tissue [27] was studied by scRNAseq under basal and β3-adrenergic stimulation conditions (CL), two major adipose stem cell (ASC) populations were identified in both adipose depots [33]. CL treatment induced the formation of proliferating and differentiating ASCs, which showed an enriched expression of genes involved in early adipogenesis. These results indicate that an adipogenic trajectory involving proliferation and differentiation is dependent on the treatment [33] of β3-adrenergic receptor activation, thus linking sympathetic tone to cellular formation. Taken together, the study indicates that β3-adrenergic receptor activation regulates the cellular plasticity of brite/beige adipocytes, and also induces the proliferation and differentiation of specific APCs to the brite/beige lineage [33]. In light of the studies that identified brite/beige APCs in the visceral adipose tissue [31], it seems that cellular heterogeneity of APCs, which is the basis for the formation of brite/beige cells in AT, is not necessarily limited to specific depots, but is a unique feature of different adipose depots. This, in turn, would imply that the failure to observe browning in certain depots might be due to reasons other than cellular composition, such as differences in innervation or possibly other inhibitory mechanisms caused by currently unidentified cell populations.

## Heterogeneity of mature adipocytes in adipose tissue

Adipocytes are the primary constituents of adipose tissue. However, due to the size and fragility of adipocytes, less research utilizing single cell/nucleus RNA approaches has been performed on the mature adipocytes. One study reported that snRNAseq sequencing conducted on the floating fraction of inguinal adipose tissue led to the identification of fourteen subpopulations of adipocytes. Among these, one cluster of cells was characterized by a high expression of ADRB3 and a robust expression of thermogenic genes [34], which can be negatively regulated by IL10 secreted from lymphocytes.

To isolate pure adipocyte nucleus fractions, a study from our lab utilized transgenic mice that expressed nuclear-targeted red fluorescent protein under the Adipoq promoter. Five subtypes of adipocytes in the brown adipose tissue were deconvoluted at room temperature, thermoneutrality, and cold exposure. A novel adipocyte subtype functioning as a paracrine cell was identified; this could regulate the activity of brown adipocytes through acetate-mediated adaptation of thermogenic capacity [35].

Another research study focused on elucidating the dynamic change of adipocytes within the epididymal adipose depot, using snRNAseq in lean and HFD-induced obese mice. Three subpopulations of adipocytes were characterized, differentiating lipogenic adipocytes, lipid-scavenging adipocytes, and stressed lipid-scavenging adipocytes in lean mice [36]. Interestingly, during HFD, differentiating lipogenic adipocytes disappeared almost completely, while the number of stressed lipid-scavenging adipocytes increased, potentially explaining the decrease in lipogenic capacity previously reported in obese rodents and humans [37].

Taken together, these studies provide new insights into the regulation of thermogenesis via the crosstalk of different adipocyte populations and suggest that the brite/beige/brown adipocyte thermogenic capacity might be regulated by secreted factors such as cytokines or metabolites.

## Prospect and limitation of scRNA seq and snRNA seq

Despite the unprecedented resolution that scRANseq has provided, it has certain limitations. scRNAseq cannot, for example, be applied to mature adipocytes due to their large size and fragile features. Therefore, the heterogeneity of mature adipocytes, especially white adipocytes, remains poorly understood. In a conceptual trade-off, snRNAseq offers an alternative to circumvent the shortcoming of scRNAseq, although with a compromised depth and the Janus-faced assumption that the cellular profile is equally reflected in nuclear RNA dynamics. One simple way to obtain adipocyte nuclei is to extract them from the floating adipocyte fraction. This approach, however, is problematic, for it has been shown that smaller adipocytes with a low lipid content might sediment, thus skewing the analysis towards large lipid-filled cells [38]. In addition, some SVF cells have shown to be co-isolated within the floating fraction. As a result, it is almost impossible to capture pure adipocyte nuclei utilizing such an approach. An alternative is to label adipocyte nuclei and to isolate said nuclei by sorting [29]. However, this method introduces possible biases based on the degree of the fluorescent protein expression. Therefore, in future investigations, a combination of technologies might be required to elucidate the heterogeneity of adipocytes in different depots under differing conditions.

The annotation of cells in the analysis of snRNAseq and scRNAseq data is of particular concern. Although some research has been carried out on unraveling cell subtypes in adipose tissue, only a small database of adipose tissue-based cell types has been published. One should be careful to define and annotate cell types when lacking a reliable reference database.

Lastly, it should be noted that, although scRNAseq provides thorough amounts of information on the identity and transcriptional landscape of newly identified subpopulations of cells, it cannot replace the comprehensive functional validation of each cell cluster. Therefore, to explain the state, identity, and function of these newly identified types of cells, cell identity data needs to be complemented with *in vitro* and *in vivo* functional experimentation. Thus, knocking out functional constituents of different populations of cells, could be an effective strategy to study the functionality of a specific cell type, *in vivo*. Furthermore, specific cell populations can be marked by diphtheria toxin receptor and can thus be conditionally ablated by diphtheria toxin. On the other hand, *in vitro* differentiation of different types of cells, followed by *in vivo* transplantation can be an informative approach towards elucidating the cells’ adipogenic potential. Lastly, different types of APCs and their developmental trajectories can be studied by Cre-Loxp-reporter based systems coupled to single cell omics-based lineage tracing. A combination of all these functional approaches will hopefully offer a toolbox to generate unprecedented insight into adipose tissue heterogeneity and functionality [39, 40].

## Abbreviations

SVF: Stromal vascular fraction
APCs: Adipogenic progenitor cells
UCP1: Uncoupling protein 1
scRNAseq: Single cell RNA sequencing
snRNAseq: Single nucleus RNA sequencing
PP1-3: Lin- progenitor cells of population 1-3from inguinal adipose tissue
FIP: Fibro-inflammatory progenitors
hP1-5: Human adipogenic progenitor cells Population 1–5
T2D: Type 2 diabetes
CL: CL316243 (β3-adrenergic receptor agonist)
ASC: Adipose stem cell
